# Caffeine Decreases Hepcidin Expression to Alleviate Aberrant Iron Metabolism under Inflammation by Regulating the IL-6/STAT3 Pathway

**DOI:** 10.3390/life12071025

**Published:** 2022-07-10

**Authors:** Zhong-Da Li, Meng-Yu Geng, Song-Rui Dou, Xuan Wang, Zi-Han Zhang, Yan-Zhong Chang

**Affiliations:** Ministry of Education Key Laboratory of Molecular and Cellular Biology, The Key Laboratory of Animal Physiology, Biochemistry and Molecular Biology of Hebei Province, College of Life Sciences, Hebei Normal University, No. 20 Nan’erhuan Eastern Road, Shijiazhuang 050024, China; lzd1995345@163.com (Z.-D.L.); baiexueshi20010305@163.com (M.-Y.G.); dousongrui200107@163.com (S.-R.D.); wangxuan4158@163.com (X.W.); zhangzizhang170524@163.com (Z.-H.Z.)

**Keywords:** caffeine, hepcidin, inflammation, IL-6, STAT3, iron metabolism

## Abstract

Caffeine is well-known as a psychostimulant, and it can also be beneficial in numerous diseases such as diabetes and different types of cancer. Previous studies have shown that caffeine can have a protective role in bacterial infection-induced inflammation and hyperoxia-mediated pulmonary inflammation. Hepcidin, which is regulated by the IL-6/STAT3 inflammation pathway, is a peptide hormone that maintains systemic iron homeostasis. We hypothesized that caffeine’s effects on inflammation may also influence hepcidin production and therefore systemic iron metabolism. To this end, we treated 2-month-old mice with caffeine by daily intragastric administration for 7 days, administering intraperitoneal LPS after the final caffeine treatment. Twelve hours after LPS treatment the mice were euthanized, and tissues were collected. We found that caffeine decreased hepatic hepcidin expression and attenuated LPS-induced hepatic hepcidin overexpression. IL-6 expression and STAT3 phosphorylation were also reduced upon caffeine administration. Additionally, hepatic and splenic FPN1 levels increased after caffeine treatment, leading to lower iron levels in liver and spleen tissues and higher iron levels in serum. Caffeine also prevented the increase in spleen weight and decrease in body weight after LPS treatment. Together, our findings suggest that caffeine decreases hepcidin expression via inhibiting inflammation and the activation of the IL-6/STAT3 pathway, thus presenting an attractive, potential therapeutic for the treatment of anemia of inflammation.

## 1. Introduction

Caffeine is an alkaloid belonging to the methylxanthines family, which is common in drinks, such as coffee and tea, and functions as a psychostimulant. Daily consumption of coffee can reduce the risk of several diseases, including Type 2 diabetes, Parkinson’s disease, cirrhosis, leukaemia, liver cancer, chronic liver disease and others [1]. Caffeine enhances cholesterol clearance by blocking SREBP2-induced PCSK9 expression in the liver [2]. Regular coffee consumption is also beneficial to cardiometabolic disease and cardiovascular health [3]. Thus, caffeine is a compound deserving of additional exploration in health care.

Iron, an essential element of life, is the second most abundant metal in Earth’s crust. This metal plays an important role in many physiological functions, such as cellular respiration, DNA replication and repair, and oxygen transport [4,5]. It follows that iron deficiency can cause anaemia and/or interfere with DNA repair, leading to developmental defects, especially in children [6]. However, iron overload can also affect normal physiological functions. Hereditary hemochromatosis (HH), an accumulation of systemic iron due to inherited defects in iron sensing, can lead to severe iron accumulation in organs when poorly managed. Cellular iron overload can lead to Fenton chemistry and oxidative stress, resulting in increased apoptosis and ferroptosis [5,7,8]. Thus, iron metabolism is a homeostatic balancing act of maintaining sufficient iron for the metal’s vital functions while avoiding the harmful effects of deficiency or excess catalytic metal. Liver-derived hepcidin, which can modulate cellular FPN1 (ferroportin 1; iron export protein) levels, is a master regulator of systemic iron balance [9].

Anaemia of inflammation (AI) is an anaemia caused by inflammation-mediated functional iron deficiency. Under inflammation, the IL-6/STAT3/hepcidin/FPN1 pathway is activated. Elevated hepcidin inhibits iron efflux from cells into the blood by binding to FPN1, which leads to FPN1 ubiquitination and degradation. The decrease in the cellular efflux of iron from the cells of the reticuloendothelial system and the duodenal enterocytes that normally supply iron for erythropoiesis results in anaemia [9,10,11]. Thus far, hepcidin is the most important target for AI treatment. The inhibition of hepcidin expression by drug therapy can alleviate serum iron deficiency and anima [10,12]. However, the methods of drug treatment are limited to transfusion and intravenous injection [10,12], and frequent punctures are very painful for patients. Caffeine has been shown to repress *Pseudomonas aeruginosa* infection-mediated inflammation by inhibiting miR-301b [13]. In *Listeria monocytogenes*-infected mice, leukocyte infiltration and the levels of cytokines were reduced after caffeine administration [14]. Moreover, hyperoxia-mediated pulmonary inflammation in rat pups was mitigated by the intraperitoneal injection of caffeine [15]. These reports are suggestive of an anti-inflammatory effect of caffeine. In addition, caffeine can suppress the symptoms of anaemia [16,17]. Despite these findings, there have been no descriptions of the mechanisms of caffeine’s effects in the regulation of body iron metabolism. We hypothesized that caffeine regulates the expression of hepcidin in the liver by affecting the inflammatory signalling pathway, thereby regulating the imbalance of iron metabolism caused by inflammation.

In our present study, we treated mice with caffeine by intragastric administration for a week, after which the animals were injected with LPS. Twelve hours later, the mice were euthanized, and the organs were collected. The caffeine-treated group exhibited decreased inflammation, reduced hepcidin expression, lower hepatic iron levels, lower splenic iron levels and elevated serum iron levels. In addition, caffeine treatment prevented the weight drop normally observed with LPS treatment. These data provide additional support for the anti-inflammatory activity of caffeine and its potential as a treatment for AI.

## 2. Materials and Methods

### 2.1. Animals

Two-month-old C57BL/6N mice were purchased from the Beijing Vitalstar Biotechnology Company. All animal experiments were approved by the Animal Ethics Committee of Hebei Normal University and by the Animal Care and Use Committee of the Hebei Science and Technical Bureau, China. All mice were housed with free access to food and water under a 12 h light/dark cycle at 21 ± 2 °C. The mice were divided into 4 groups: (1) Sal + Sal (saline intragastric administration and saline intraperitoneal injection); (2) Sal + LPS (saline intragastric administration and 10 mg/kg body weight LPS intraperitoneal injection); (3) Caf + Sal (100 mg/kg body weight caffeine intragastric administration and saline intraperitoneal injection); (4) Caf + LPS (100 mg/kg body weight caffeine intragastric administration and 10 mg/kg body weight LPS intraperitoneal injection). The mice were treated with saline or caffeine once a day for 7 days via intragastric administration followed by LPS intraperitoneal injection. After 12 h of LPS injection, the mice were euthanatized with 0.8% pentobarbital sodium, and then the blood was removed by perfusion with pre-cooled saline. Finally, tissue and organs were harvested for subsequent experiments.

### 2.2. Quantitative Real-Time PCR (qPCR)

The RNA extraction procedure was described previously [18]. Total RNA extracted from the liver with TRIzol Reagent (Invitrogen, Thermo Fisher Scientific-CN, Shanghai, China) was used for cDNA synthesis using a PrimeScript™ RT reagent kit (RR037A, Takara Biomedical Technology Co., Ltd., Beijing, China). Quantitative real-time PCR was performed using UltraSYBR Mixture (CW0957L, Cwbio Biotechnology Limited Company, Beijing, China) and a Bio-Rad CFX 96 detection system (Bio-Rad, Singapore). The primers used are shown in Table 1.

### 2.3. Western Blot Analysis

Protein extraction from liver and spleen tissue was performed with RIPA buffer (50 mM Tris-HCl pH 7.4, 150 mM NaCl, 1% NP40, 0.1% SDS) containing protease inhibitor cocktail tablets (68298, Roche Applied Science, Mannheim, Germany) and PMSF (329-98-6, Solarbio^®^, Beijing, China). Protein expression was analyzed by western blot analysis using a previously described procedure [19,20]. The primary antibodies used were shown as follows: FTL (rabbit anti-mouse, 1:5000; ab109373, Abcam, San Francisco, CA, USA), FTH (rabbit anti-mouse, 1:10,000; ab183781, Abcam, San Francisco, CA, USA), FPN1 (rabbit anti-mouse, 1:10,000; MTP11-S, ADI, San Antonio, TX, USA), TfR1 (mouse anti-mouse, 1:10,000; 13-6800, Invitrogen, Carlsbad, CA, USA), β-actin (mouse anti-mouse, 1:10,000; CW0096, Cwbio Biotechnology Limited Company, Beijing, China), p-STAT3 (rabbit anti-mouse, 1:5000; 9145S, CST, St. Louis, MA, USA), STAT3 (rabbit anti-mouse, 1:5000; 12640S, CST), and GAPDH (mouse anti-mouse, 1:10,000; 60004-1-lg, Proteintech, Rosemont, PA, USA). After washing with TBST (pH 7.6, 137 mM NaCl, 0.1% Tween-20), the membranes were incubated with HRP-conjugated goat anti-mouse IgG (H + L) (1:10,000; RS0001, Immunoway, Plano, TX, USA) or HRP-conjugated goat anti-rabbit IgG (H + L) (1:10,000; RS0002, Immunoway, Plano, TX, USA) for 1.5 h at room temperature. After washing three times in TBST, proteins were detected by chemiluminescence (ECL).

### 2.4. Inductively Coupled Plasma Mass Spectrometry (ICP–MS)

Splenic iron content was determined by ICP–MS as described previously [21]. Spleen tissue samples were treated with 69% HNO_3_ at room temperature overnight, then incubated at 90 °C for 20 min, with a further incubation in hydrogen peroxide (30%; Merck, Shanghai, China; equivalent volume of HNO_3_) at 70 °C for 15 min, followed by evaporation to dryness at 100 °C for 9 h. Finally, deionized water (≥18.2 MΩ) was added. The splenic iron content was detected using an ICP–MS spectrometer (Agilent 7700; Agilent Technologies, Santa Clara, CA, USA).

### 2.5. Measurement of Serum Iron

Blood samples were obtained from mouse eye sockets and then centrifuged at 2000 *g* at 4 °C for 20 min, and then the serum was collected as previously described [22]. Serum iron contents were detected with a serum iron measurement reagent kit (A039-1-1, Nanjing Jiancheng Bioengineering Institute, Nanjing, China) according to the manufacturer’s instructions. These results can be detected by spectrophotometer (Synergy H4, BioTek, Santa Clara, CA, USA) at the absorbance of 520 nm.

### 2.6. Enzyme Linked Immunosorbent Assay (ELISA)

The contents of IL-6 in serum were assayed with ELISA kits (EIA-2019, Elias Biotech, Shanghai, China) according to the manufacturer’s instructions. Briefly, blood samples were obtained from mouse eye sockets and then centrifuged at 2000 *g* at 4 °C for 20 min, and then the serum was collected. A volume of 50 μL of the serum was prepared for measuring IL-6. These measurements can be detected by spectrophotometer (Synergy H4, BioTek, Santa Clara, CA, USA) at the absorbance of 450 nm.

### 2.7. Statistical Analysis

Statistical analyses were performed using GraphPad Prism 6.0. The data are presented as the mean ± SD. The analysis of comparison between the two groups was assessed using a two-tailed Student’s *t*-test. The significance of the differences between the two groups was determined by the *p*-value of Student’s *t*-test. Differences were considered statistically significant when the *p*-value was less than 0.05 (*p* < 0.05); * *p* < 0.05, ** *p* < 0.01, *** *p* < 0.001, Sal + LPS vs. Sal + Sal; ^&^ *p* < 0.05, ^&&^ *p* < 0.01, ^&&&^ *p* < 0.001, Caf + Sal vs. Sal + Sal; ^#^ *p* < 0.05, ^##^ *p* < 0.01, ^###^ *p* < 0.001, Caf + LPS vs. Sal + LPS; ^$^ *p* < 0.05, ^$$^ *p* < 0.01, ^$$$^ *p* < 0.001, Caf + LPS vs. Caf + Sal.

## 3. Results

### 3.1. LPS-Induced Elevated Hamp Expression Was Alleviated by Caffeine

**Figure 1:**
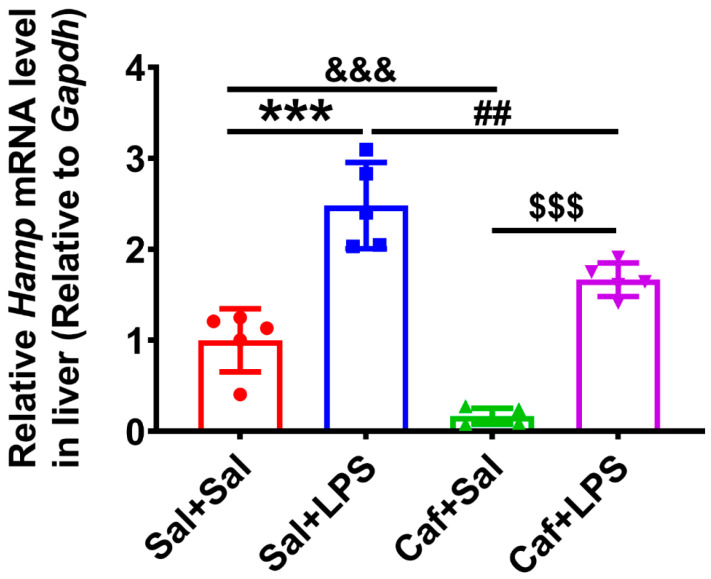
Hepatic *Hamp* mRNA levels as measured by qPCR (*n* = 5). *** *p* < 0.001, Sal + LPS vs. Sal + Sal; ^&&&^ *p* < 0.001, Caf + Sal vs. Sal + Sal; ^##^ *p* < 0.01, Caf + LPS vs. Sal + LPS; ^$$$^ *p* < 0.001, Caf + LPS vs. Caf + Sal.

After 7 consecutive daily intragastric administrations of caffeine, we treated the mice with LPS for 12 h, and then extracted their livers for experiments. We detected the expression of *Hamp* (the gene of hepcidin) and found that the expression of *Hamp* was significantly increased after LPS treatment (Sal + LPS vs. Sal + Sal, Caf + LPS vs. Caf + Sal) (Figure 1). However, in the caffeine-administered groups (Caf + Sal and Caf + LPS), the *Hamp* expression was significantly decreased (Caf + Sal vs. Sal + Sal, Caf + LPS vs. Sal + LPS) (Figure 1). These results suggest that caffeine reduces *Hamp* levels and prevents the inflammation-induced elevation of *Hamp* in the liver.

### 3.2. Caffeine Suppressed LPS-Induced Upregulated Il6 Expression and STAT3 Phosphorylation

**Figure 2:**
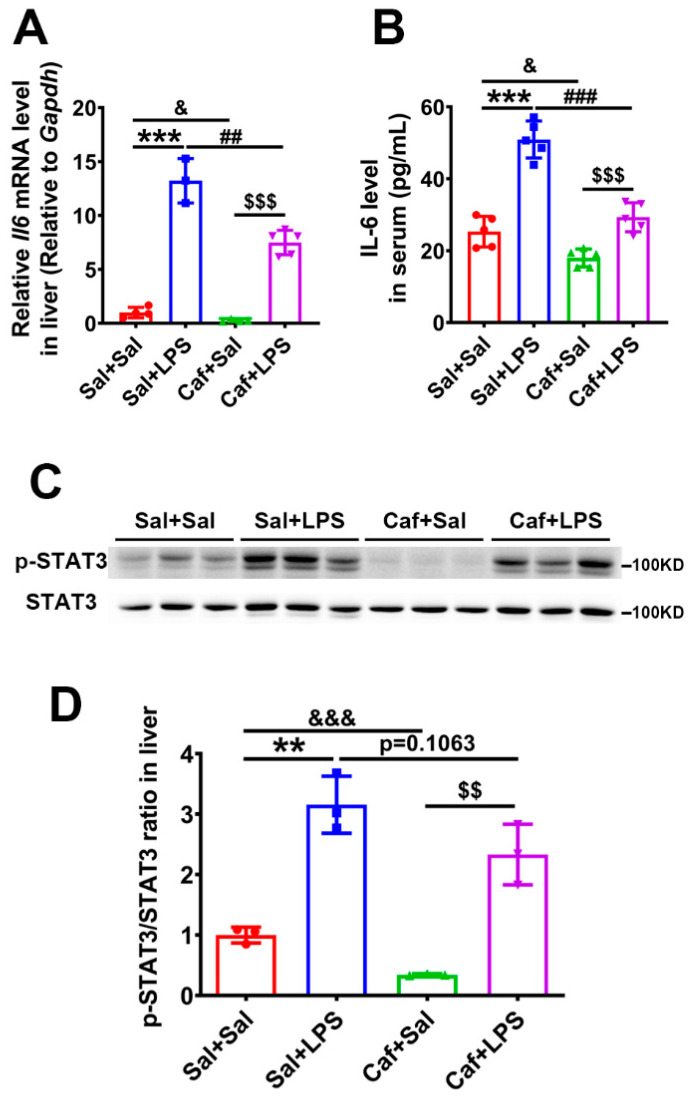
Expression of *Il6* mRNA and phosphorylation levels of STAT3 in liver. (**A**) qPCR analysis of *Il6* mRNA in liver tissue (*n* = 3 for Sal + LPS, *n* = 4 for Sal + Sal and Caf + Sal, *n* = 5 for Caf + LPS). (**B**) ELISA analysis of IL-6 levels in serum (*n* = 5). (**C**) Western blot analysis of phosphorylated STAT3 and total STAT3 levels in liver tissue. (**D**) The ratio of p-STAT3/STAT3 quantified from the data in chart B (*n* = 3). ** *p* < 0.01, *** *p* < 0.001, Sal + LPS vs. Sal + Sal; ^&^ *p* < 0.05, ^&&&^ *p* < 0.001, Caf + Sal vs. Sal + Sal; ^##^ *p* < 0.01, ^###^ *p* < 0.001, Caf + LPS vs. Sal + LPS; ^$$^ *p* < 0.01, ^$$$^ *p* < 0.001, Caf + LPS vs. Caf + Sal.

Hepcidin expression is regulated by the IL-6/STAT3 pathway. IL-6 has been shown to induce marked expression of hepcidin that can be blocked by STAT3 siRNA [23]. Therefore, we examined the IL-6 and STAT3 phosphorylation levels in liver tissue. After LPS injection, the expression of the *Il6* (IL-6 gene) in the liver was upregulated ~13-fold in the Sal + LPS group compared with the Sal + Sal group and ~28-fold in the Caf + LPS group compared with the Caf + Sal group. In the groups administered caffeine (Caf + Sal and Caf + LPS), the expressions of *Il6* were significantly lower (Caf + Sal vs. Sal + Sal, Caf + LPS vs. Sal + LPS) (Figure 2A). The IL-6 levels in serum were also obviously lower after caffeine administration (Caf + Sal vs. Sal + Sal, Caf + LPS vs. Sal + LPS) (Figure 2B). Western blot analysis verified that the phosphorylation of STAT3 (p-STAT3/STAT3 ratio) in the liver was significantly increased after LPS treatment (Sal + LPS vs. Sal + Sal, Caf + LPS vs. Caf + Sal) but decreased in the caffeine-administered group (Caf + Sal vs. Sal + Sal, Caf + LPS vs. Sal + LPS) (Figure 2C,D). These results indicate that caffeine may inhibit IL-6 expression to attenuate the LPS/IL-6/STAT3 pathway, leading to decreased hepcidin production in the liver.

### 3.3. Caffeine Decreased Iron Levels and Attenuated LPS-Mediated Iron Accumulation in the Liver

**Figure 3:**
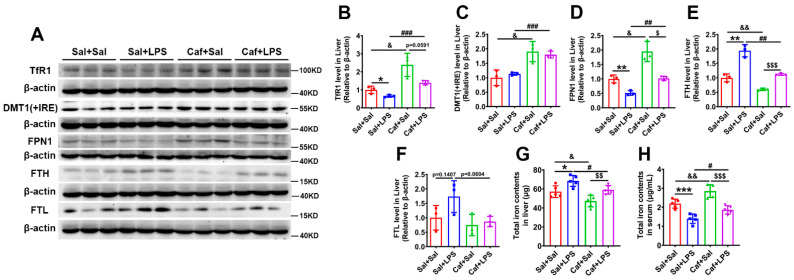
Iron metabolism-related protein expression in the liver. (**A**) Western blot analysis of TfR1, DMT1(+IRE), FPN1, FTH and FTL levels in the liver. (**B**–**F**) Quantification of the results of the experiment shown in chart A (*n* = 3). (**G**) Total iron contents in the liver as detected by ICP-MS (*n* = 5). (**H**) Total iron contents in serum (*n* = 5). * *p* < 0.05, ** *p* < 0.01, *** *p* < 0.001, Sal + LPS vs. Sal + Sal; ^&^ *p* < 0.05, ^&&^ *p* < 0.01, Caf + Sal vs. Sal + Sal; ^#^ *p* < 0.05, ^##^ *p* < 0.01, ^###^ *p* < 0.001, Caf + LPS vs. Sal + LPS; ^$^ *p* < 0.05, ^$$^ *p* < 0.01, ^$$$^ *p* < 0.001, Caf + LPS vs. Caf + Sal.

Hepcidin regulates iron homeostasis via decreasing FPN1 levels, so elevated plasma levels of hepcidin block iron export from cells into the blood, leading to a deficiency in iron availability for erythropoiesis and iron retention in the cells that recycle (hepatic and splenic macrophages), transport (enterocytes) and store (hepatocytes) the metal [4,9,24,25]. Thus, we hypothesized that caffeine administration would lead to a decrease in iron levels in the liver. To test this, we examined the levels of proteins related to iron metabolism by western blot analysis (Figure 3A). TfR1 (transferrin receptor 1, the gateway to cellular iron uptake), DMT1(+IRE) (divalent metal transporter 1 with IRE; cellular iron import protein) and FPN1 (ferroportin 1; iron export protein) levels were all increased after caffeine administration (Figure 3B–D). After caffeine administration, FTH (ferritin heavy chain; iron storage protein) levels were significantly decreased (Figure 3E), and FTL (ferritin light chain; iron storage protein) levels were unchanged (Figure 3F). In addition, caffeine administration significantly alleviated LPS-induced TfR1 and FPN1 downregulation (Figure 3B,D) and FTH upregulation (Figure 3E). The regulation of TfR1, DMT1(+IRE), FPN1, FTH and FTL are all under the control of the IRE–IRP regulation system, in which uncommitted cellular iron levels directly switch on and off the ability of iron regulatory proteins (IRPs) to bind to iron-responsive elements (IREs) located in the regulatory regions of the mRNAs that encode proteins of iron metabolism [9,25]. Proteins of iron uptake, such as TfR1 and DMT(+IRE), increase when cellular iron levels are low, and IRPs bind to IREs in the 3′ UTRs (untranslated regions) of the respective mRNAs, while IRP binding to the 5′ UTRs of mRNAs that encode proteins of iron storage (ferritin) and export (FPN1) blocks protein translation.

To confirm whether iron contents were decreased by caffeine, we used ICP–MS to detect hepatic iron contents, and we found total hepatic iron contents were decreased in caffeine administration groups (Figure 3G) (Caf + Sal vs. Sal + Sal, Caf + LPS vs. Sal + LPS). The iron contents in serum were on the contrary increased after caffeine administration (Figure 3H) (Caf + Sal vs. Sal + Sal, Caf + LPS vs. Sal + LPS). Our results suggest that caffeine blocks hepcidin expression, leading to a stabilization of FPN1 levels that in turn decreases intracellular iron through exporting the metal, with the serum iron increased. The decreased intracellular iron levels then lead to IRP activation, which increases the levels of TfR1 and DMT(+IRE) and decreases FTH levels [9,25]. Then we confirmed that, congruent with the results of the molecular assays, 7 days of caffeine administration inhibited LPS-mediated iron accumulation in the liver.

### 3.4. Caffeine Reduced Iron Content in the Spleen after LPS Treatment

**Figure 4:**
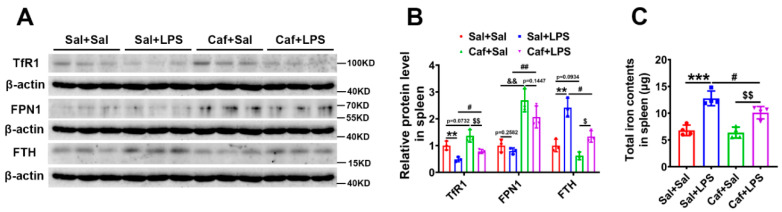
Splenic iron status. (**A**) Western blot analysis of TfR1, FPN1, and FTH levels in the spleen. (**B**) Quantification of the results of the experiment shown in chart A (*n* = 3). (**C**) Total iron contents in the spleen as detected by ICP-MS (*n* = 4). ** *p* < 0.01, *** *p* < 0.001, Sal + LPS vs. Sal + Sal; ^&&^ *p* < 0.01, Caf + Sal vs. Sal + Sal; ^#^ *p* < 0.05, ^##^ *p* < 0.01, Caf + LPS vs. Sal + LPS; ^$^ *p* < 0.05, ^$$^ *p* < 0.01, Caf + LPS vs. Caf + Sal.

We were curious about how splenic iron content was affected by caffeine administration. Similar to the results in liver tissue, western blot analysis revealed an increase in TfR1 and a decrease in FTH in the Caf + LPS group compared with Sal + LPS (Figure 4A,B). FPN1 expression was also markedly increased (Caf + Sal vs. Sal + Sal, Caf + LPS vs. Sal + LPS) (Figure 4A,B). The total iron content in the spleen measured by ICP–MS showed that caffeine administration prevented the LPS-induced iron overload (Figure 4C). These findings suggest that caffeine can also decrease iron levels in the spleen via stabilizing FPN1 levels.

### 3.5. Caffeine Alleviated LPS-Induced Splenomegaly and Weight Loss

**Figure 5:**
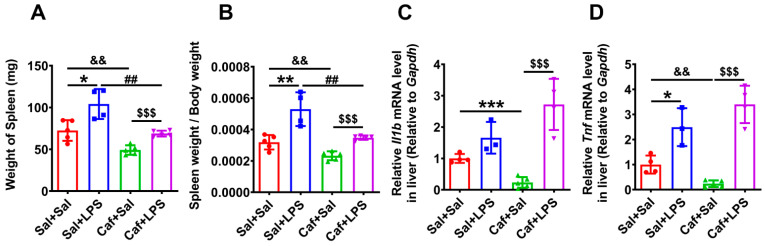
Splenic weights and the expression of hepatic *Il1b* and *Tnf*. (**A**) Spleen weights. (**B**) Spleen/body weight ratios (*n* = 4 for Sal + LPS, *n* = 5 for Sal + Sal, Caf + Sal and Caf + LPS). (**C**,**D**) qPCR analysis of *Il1b* and *Tnf* mRNA level in liver tissue (*n* = 3 for Sal + LPS, *n* = 4 for Sal + Sal and Caf + LPS, *n* = 5 for Caf + Sal). * *p* < 0.05, ** *p* < 0.01, *** *p* < 0.001, Sal + LPS vs. Sal + Sal; ^&&^ *p* < 0.01, Caf + Sal vs. Sal + Sal; ^##^ *p* < 0.01, Caf + LPS vs. Sal + LPS; ^$$$^ *p* < 0.001, Caf + LPS vs. Caf + Sal.

**Figure 6:**
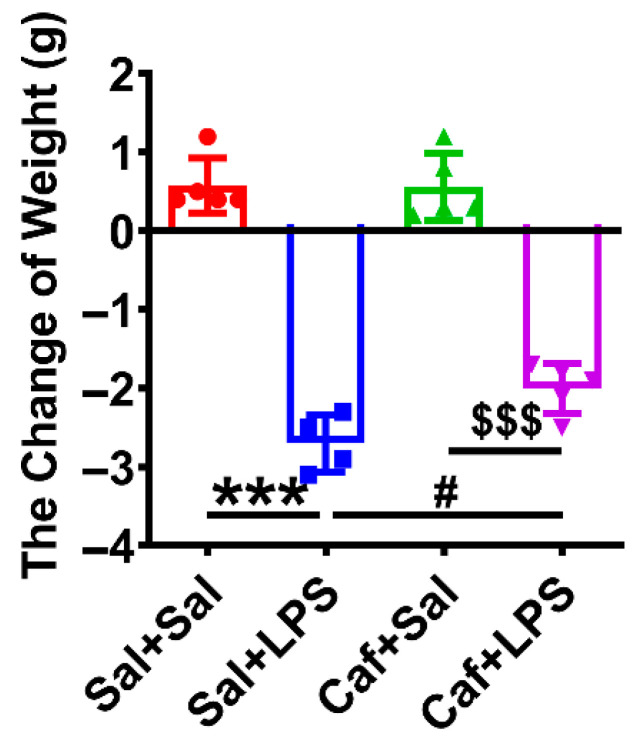
Changes in body weight before and 12 h after LPS injection (*n* = 4 for Sal + LPS, *n* = 5 for Sal + Sal, Caf + Sal and Caf + LPS). *** *p* < 0.001, Sal + LPS vs. Sal + Sal; ^#^ *p* < 0.05, Caf + LPS vs. Sal + LPS; ^$$$^ *p* < 0.001, Caf + LPS vs. Caf + Sal.

Twelve hours after LPS injection, we euthanized the mice and weighed the spleens. We found that the spleen weight and the ratio of spleen to body weight were significantly increased 12 h after LPS injection, while caffeine alleviated this effect (Figure 5A,B). Expression of *Il1b* (the gene of IL-1β) and *Tnf* (the gene of TNF-α) was significantly decreased in the Caf + Sal group compared with the Sal + Sal group but not altered significantly in the Caf + LPS group compared with the Sal + LPS group (Figure 5C,D). Thus, caffeine administration may not prevent the induction of IL-1β and TNF-α expression by LPS. We also measured the body weight of each mouse and found that LPS induced a significant loss in weight that could be prevented by caffeine administration (Figure 6).

## 4. Discussion

Caffeine has been shown to exert various health benefits, including the prevention of a diverse set of diseases including some types of cancer [1]. Bacterial infection and hyperoxia-mediated inflammation can also be alleviated by caffeine [13,14,15]. Inflammation can elicit hepcidin overexpression via the IL-6/STAT3 pathway [9,22]. However, there were few studies on the relationship of caffeine and hepcidin. Here, we first found that caffeine can affect hepatic hepcidin expression to prevent inflammation-induced hepatic hepcidin overexpression.

There are various pathways involved in hepcidin regulation, including TfR2/HFE, BMP/SMAD, and IL-6/STAT3 [4,9,26]. Bone morphogenetic protein 6 (BMP6) can bind to the BMP receptor in conjunction with haemojuvelin (HJV), resulting in SMAD phosphorylation in downstream signalling, which stimulates hepcidin expression. SMAD signalling can also be regulated by transferrin receptor 2 (TfR2) and human haemochromatosis protein (HFE). Therefore, the BMP/SMAD pathway plays an important role in the regulation of hepcidin. In addition, under the condition of inflammation, more IL-6 binds to IL-6R, resulting in JAK/STAT3 activation and hepcidin transcription. The IL-6/STAT3 pathway is independent of the BMP/SMAD pathway in hepcidin regulation [4]. In the present study, LPS enhanced IL-6 expression, while caffeine abrogated the LPS induction of IL-6 expression. We therefore focused on the IL-6/STAT3 pathway and found that STAT3 phosphorylation was also downregulated. In addition, the LPS-induced increase in splenic weight was lower in the LPS plus caffeine group. However, under LPS treatment, caffeine did not affect the IL-1β and TNF-α levels in the liver. These results revealed that caffeine reduced hepcidin expression by decreasing IL-6 expression and STAT3 phosphorylation.

The iron export protein FPN1 is inhibited by hepcidin, leading to iron retention in the cells responsible for donating the metal to transferrin (Tf) in the plasma [18,23,24,27,28]. After caffeine administration, hepatic FPN1 expression increased, with a concomitant decrease in liver iron levels. The total iron levels in the whole spleen upon LPS injection also decreased after caffeine administration, again with FPN1 markedly elevated compared with the Sal + LPS group. Therefore, iron export into serum and iron contents in serum both increased. Taken together, our data demonstrate that caffeine can stimulate FPN1 levels by decreasing hepcidin expression, resulting in increased iron export from the liver and spleen and increased serum iron, which could improve the availability of the metal for erythropoiesis.

Inflammation, especially chronic inflammation, often causes anaemia (AI) as a secondary manifestation of chronic disease. Treating the inflammation can reverse this anaemia symptom, but such treatment is not always feasible. Therefore, strategies to directly treat the anaemia would present attractive alternatives. To treat AI, potential therapies include erythrocyte transfusions, erythropoiesis-stimulating agents (ESAs; erythropoietin and its mimics) and sometimes iron supplementation. There are also experimental treatments for AI, including those that target cytokines affecting the hepcidin–FPN1 axis and other molecular regulators upstream of hepcidin expression [10,12]. Some of these potential treatments have progressed from animal models to clinical trials [10,12]. In some very severe inflammatory diseases, the efficient inhibition of anti-IL-6 was achieved with pharmaceutical agents. Drugs that inhibit the IL-6 pathway may also be effective in AI treatment. As discussed above, the therapeutic strategies for AI have mainly concentrated on the inhibition of IL-6, IL-6R, hepcidin, BMP and TfR2 expression by drug administration [12,29,30,31]. However, the methods of administration of these drugs are limited to intravenous injection and intravenous transfusion [10,12], which are painful for patients. Our findings suggested that caffeine can decrease hepcidin expression in the face of inflammation, improve iron export from the liver and spleen and increase the iron content of serum, which represents a potentially new treatment strategy for AI. It is possible that caffeine will become a new drug in AI prevention and even therapy. We hope that AI patients will experience positive effects just from drinking a cup or two of coffee daily.

## 5. Conclusions

Caffeine inhibits hepatic hepcidin expression by decreasing IL-6 levels and the phosphorylation of STAT3 to diminish hepatic and splenic iron contents, with serum iron contents increased. Through this mechanism, caffeine alleviates LPS-induced hepcidin overexpression via reducing the IL-6/STAT3 pathway. Thus, caffeine treatment could be a new strategy to prevent and cure AI.

## Figures and Tables

**Table 1:** qPCR primers.

Gene	Forward	Reverse
*Gapdh*	5′-TGCACCACCAACTGCTTAGC-3′	5′-GGCATGGACTGTGGTCATGAG-3′
*Hamp*	5′-AGACATTGCGATACCAATGCA-3′	5′-GCAACAGATACCACACTGGGAA-3′
*Il6*	5′-ACCGCTATGAAGTTCCTCTC-3′	5′-CTCTGTGAAGTCTCCTCTCC-3′
*Il1b*	5′-CCAGCAGGTTATCATCATCATCC-3′	5′-CTCGCAGCAGCACATCAAC-3′
*Tnf*	5′-TACTGAACTTCGGGGTGATTGGTCC-3′	5′-CAGCCTTGTCCCTTGAAGAGAACC-3′

## Data Availability

The data presented in this study are available on request from the corresponding author.
